# Updates on the Strategies to Improve the Anti-Tumor Efficacy of Ferulic Acid

**DOI:** 10.3390/ijms27167324

**Published:** 2026-08-16

**Authors:** Tiziana Fiore, Michela Giuliano, Claudia Pellerito, Sonia Emanuele

**Affiliations:** 1 Department of Physics and Chemistry “Emilio Segrè”, University of Palermo, 90128 Palermo, Italy; tiziana.fiore@unipa.it (T.F.); claudia.pellerito@unipa.it (C.P.); 2 Department of Biological, Chemical and Pharmaceutical Sciences and Technologies (STEBICEF), University of Palermo, 90127 Palermo, Italy; michela.giuliano@unipa.it; 3 Department of Biomedicine, Neurosciences and Advanced Diagnostics (BIND), Biochemistry Building, University of Palermo, 90127 Palermo, Italy

**Keywords:** ferulic acid, tumor, cell death, nanodevices, chemical conjugates, organotin(IV) derivatives

## Abstract

Ferulic acid, a natural phenolic phytotherapeutic, which is mainly found in plant cell walls, has attracted the attention of researchers for its multiple pharmacological properties, especially for its anti-tumor potential. As an efficient antioxidant, the compound counteracts oxidative stress and modulates key molecular pathways involved in carcinogenesis. Recent studies demonstrate that ferulic acid exerts antiproliferative, pro-apoptotic, and anti-metastatic effects in various tumor models, including colon, breast, liver, and lung cancers. Mechanistically, ferulic acid affects components of prosurvival-signaling pathways such as PI3K/Akt, MAPK, and NF-κB, and stimulates programmed cell death by diverse mechanisms, including apoptosis, autophagy and ferroptosis. Furthermore, its ability to sensitize cancer cells to chemotherapeutic drugs with low toxicity to normal cells underscores its therapeutic potential. Despite promising preclinical anti-tumor activity, low water solubility and bioavailability limit its clinical use. For this reason, several attempts, ranging from chemical derivatives to nanodevices, have been made to improve ferulic bioavailability and anti-tumor efficacy. This review highlights the most significant and recent strategies to ameliorate the anticancer ability of ferulic acid in the perspective of a clinical application.

## 1. Introduction

Ferulic acid (4-hydroxy-3-methoxycinnamic acid) is a naturally occurring phenolic compound belonging to the class of hydroxycinnamic acids. From a chemical point of view, the compound structurally derives from cinnamic acid and is characterized by an aromatic benzene ring with a hydroxyl group (-OH) at the para (4-) position and a methoxy group (-OCH3) at the ortho (3-) position relative to the propenoic acid side chain. This side chain, which includes a conjugated double bond and a carboxylic acid group (-COOH), extends the electronic system of the molecule, thereby enhancing its antioxidant activity.

Ferulic acid (FA) is widely distributed throughout the plant kingdom and plays several critical roles in plant biology. It is especially abundant in cereal grains such as wheat, rice, oats, and corn, where it is predominantly located in the bran layer. Among cereals, corn bran and rice bran are particularly rich sources. Other dietary sources include fruits, vegetables, seeds, and beverages such as apples, eggplant, artichokes, tomatoes, oranges, coffee, and beer, although at lower concentrations [1,2].

In plants, FA is mainly found in the cell walls, where it is commonly esterified and bound to lignin and polysaccharides such as hemicelluloses [3]. Beyond its structural function, FA also plays a significant role in plant defense [4]. Its ability to cross-link wall components limits the spread of pathogens by creating a more impenetrable barrier [5]. Moreover, the compound exhibits antimicrobial activity by disrupting bacterial cell membranes and inhibiting key metabolic enzymes, helping to protect plants from a wide range of bacterial and fungal pathogens [6]. Additionally, its potent antioxidant properties mitigate oxidative stress by scavenging reactive oxygen species (ROS), which accumulate during environmental stressors such as UV radiation, drought, or pathogen attacks [4]. However, despite its promising antimicrobial potential, these effects occur at relatively high concentrations, thus limiting the practical application of FA as a natural antimicrobial.

Due to its multifunctional properties, FA has garnered interest beyond plant physiology, especially in the fields of human health and cosmetic science. Specifically, FA is widely known for its antioxidant properties, which account for health-promoting effects and potential therapeutic benefits. Indeed, the effects of FA have been investigated in the context of both acute and chronic conditions, including diabetes, neurodegenerative diseases, cardiovascular disorders [7,8] and especially cancer.

A growing body of evidence has highlighted the anti-tumor potential of ferulic acid [9]. Its ability to modulate key signaling pathways involved in cell proliferation, apoptosis, and oxidative stress make it a promising candidate for cancer prevention and therapy [10]. Moreover, FA has been used in combination with chemotherapeutic or anti-tumor drugs with encouraging results [11,12,13].

Nevertheless, despite these promising pre-clinical findings, the anti-cancer therapeutic application of FA remains limited, due to its low bioavailability and rapid metabolism. To overcome these limitations and maximize its therapeutic benefits, increasing attention has been directed toward the development of novel FA-conjugated molecules or nanotechnological devices to vehicle the compound. FA conjugates often exhibit enhanced stability, bioavailability, and diverse biological activities compared to free FA. They were specifically developed to improve its pharmacokinetic properties, enhance its selectivity for cancer cells and potentiate its anti-tumor efficacy, paving the way for more effective and targeted cancer treatments. Nanotechnologies have rendered FA easily available to tumor cells, overcoming the limited bioavailability and in most cases drug delivery has significantly improved the tumor targeting effects of the compound.

The aim of this review is to describe the recent advances to enhance the anti-tumor efficacy of FA focusing on its conjugates, exploring their chemical structures and biological activities. Moreover, a special focus is placed on FA delivery strategies using a plethora of nanodevices.

## 2. Anti-Tumor Action of Ferulic Acid

### 2.1. Inhibition of Cancer Cell Proliferation

Recent evidence supports a great potential of FA as a bioactive anti-tumor compound through multiple molecular mechanisms that have been widely described in the literature [14].

FA has been shown to reduce uncontrolled cell proliferation by modulating the cell cycle machinery. Indeed, the compound has been shown to induce cell cycle arrest at the G1/S or G2/M phase, depending on the tumor type [15]. Specifically, downregulation of cyclins (e.g., cyclin D1) and/or cyclin-dependent kinases (CDKs), along with upregulation of CDK inhibitors such as p21/Cip1 and p27/Kip1 has been described [16,17]. Furthermore, FA affects the mitogen-activated protein kinase/Extracellular Signal-Regulated Kinase (MAPK/ERK) and Phosphatidylinositol 3-kinase/Ak strain transforming (PI3K/Akt) pathways, which are both crucial for promoting cell growth and survival, resulting in reduced mitogenic signaling [18].

It is well known that tumor cells increase reactive oxygen species (ROS) production to promote tumor cell survival and propagation [19,20]. FA exerts potent antioxidant effects by scavenging ROS and modulating oxidative stress pathways, thereby mitigating DNA damage and mutagenesis that contribute to oncogenesis [10,21]. The compound also modulates phase I and phase II detoxifying enzymes at hepatic level, thereby enhancing cellular defense mechanisms against carcinogens [22].

The anti-tumor activity of FA is also correlated with its anti-inflammatory potential. Indeed, the compound was shown to inhibit pro-inflammatory transcription factors such as nuclear factor kappa-light-chain-enhancer of activated B cells (NF-κB) and downstream effectors like Cyclooxygenase 2 (COX-2) and inducible nitric oxide synthetase (iNOS), attenuating inflammation-driven tumor promotion [16,23]. Additionally, evidence has been provided that the compound interferes with PI3K/Akt, MAPK, and signal transducer and activator of transcription 3 (STAT3) oncogenic signaling cascades that promote cell survival, proliferation, and metastasis [16,18].

### 2.2. Ferulic Acid and Programmed Cell Death

FA has revealed significant pro-apoptotic activity in various cancer cell lines, including colon, breast, liver, and lung cancers [24]. The compound has been shown to activate both the intrinsic and extrinsic apoptotic pathways. Involvement of the intrinsic pathway has been evidenced by the release of pro-apoptotic factors from mitochondria, such as cytochrome c, with the consequent activation of caspase-9 and downstream caspase-3 [25]. Moreover, the compound has been shown to modulate the expression of key regulators of mitochondrial apoptosis including pro-apoptotic members of the B-cell lymphoma 2 (Bcl-2) family such as Bax and downregulating the anti-apoptotic Bcl-2 [26]. Concerning the apoptotic extrinsic pathway, evidence has been provided that FA upregulates Fas receptor/Fas ligand (Fas/FasL) signaling, thus contributing to the activation of caspase-8 [27].

Both in vitro and in vivo breast cancer models, the pro-apoptotic action of FA has been related with increased expression of tumor protein p53 (p53) and its transcriptional targets p21 and BAX [25,28].

Recent studies suggest that FA may also influence autophagy, a cellular catabolic process with a dual role in cancer [29]. FA appears to induce the autophagic flux in certain cancer cells by modulating AMP-activated protein kinase/mammalian target of rapamycin (AMPK/mTOR) signaling. Specifically, FA has been shown to activate AMPK, which in turn inhibits mTORC1, a master negative regulator of autophagy. This leads to increased formation of autophagosomes and upregulation of autophagy-related proteins such as microtubule-associated protein 1A/1B-light chain 3 (LC3-II) and Beclin-1 [15,30].

FA has also been shown to activate other types of cell death. For instance, despite the controversial role of the compound in ferroptosis, a type of iron-dependent programmed cell death [31], it seems that in cancer cells FA is able to promote this process. Specifically, Cao et al. have shown that FA reduces growth and invasiveness of esophageal squamous cell carcinoma by activating ferroptotic cell death [32].

**Figure 1:**
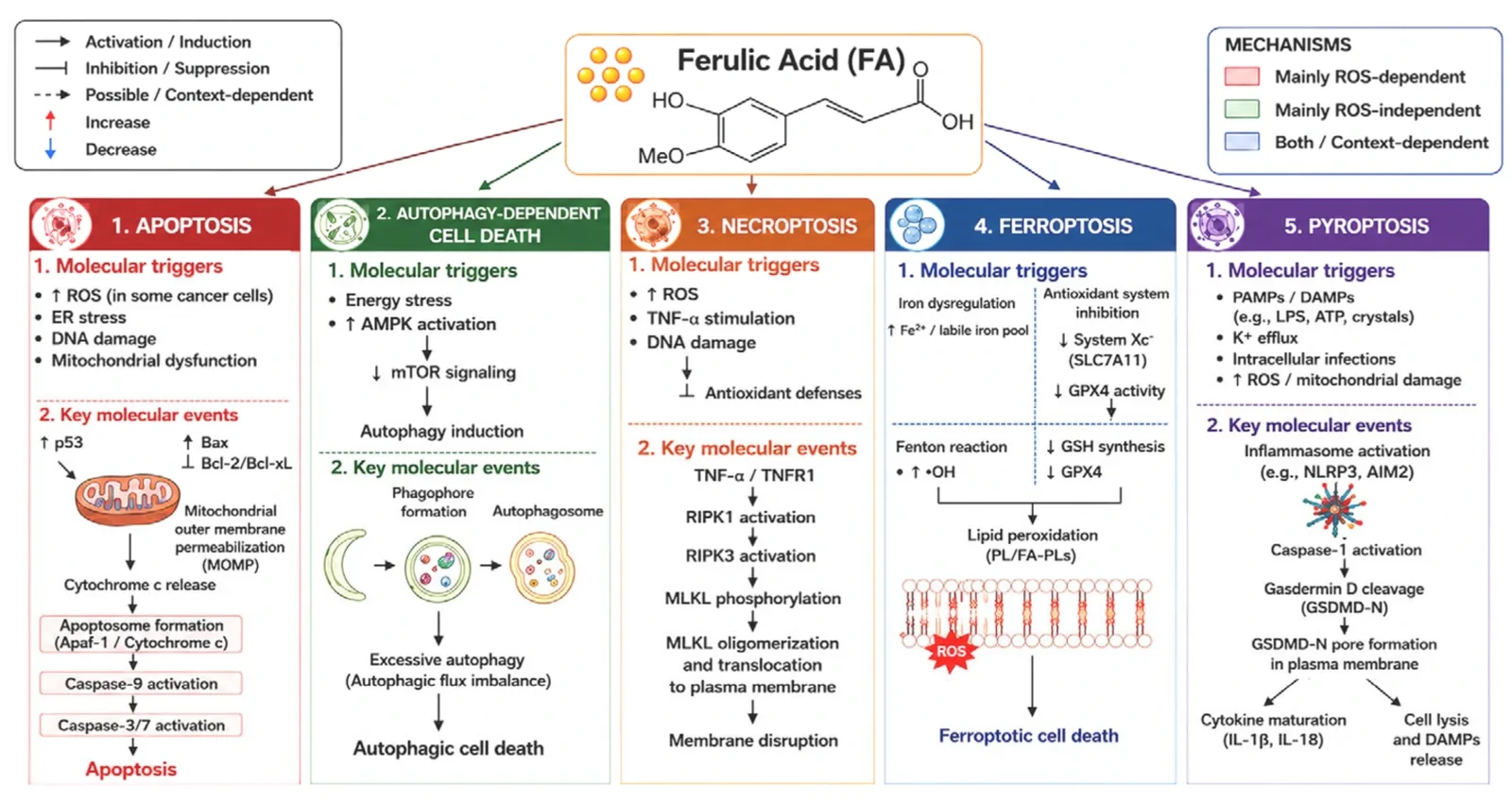
Main cell death pathways induced by ferulic acid. Conceptual illustration created by the Authors with the assistance of generative AI.

Finally, a role of FA was also attributed to a crosstalk between apoptosis and the induction of pyroptosis, a caspase 1-dependent, inflammatory type of cell death, through the ROS/Jun n-terminal kinase (JNK) pathway in lung cancer [33]. Figure 1 describes ferulic acid structure and the types of cell death induced in tumor cells.

### 2.3. Anti-Metastatic and Anti-Invasive Potential: Implication of Redox Balance

Interestingly, FA also exhibits anti-metastatic properties by inhibiting migration, invasion, and adhesion of cancer cells [14]. One proposed mechanism involves the downregulation of matrix metalloproteinases (MMPs), particularly MMP-2 and MMP-9, which are responsible for degrading extracellular matrix components and facilitating invasion [34]. FA also disrupts epithelial–mesenchymal transition (EMT) by maintaining the expression of E-cadherin and suppressing N-cadherin and vimentin, which are typically upregulated in metastatic cells. Key signaling pathways such as transforming growth factor beta/small worm phenotype-mothers against decapentaplegic (TGF-β/SMAD), wingless related integration site/beta-catenin (Wnt/β-catenin), and NF-κB which are frequently implicated in EMT and metastasis, are modulated by FA, reducing the metastatic potential of tumor cells [35,36].

It is important to highlight that the diverse anticancer activities of ferulic acid ranging from the induction of apoptosis or autophagy to the inhibition of proliferation and metastasis can be partially attributed to its dual role as an antioxidant or context-dependent pro-oxidant. In normal physiological conditions, FA primarily functions as a potent antioxidant, ROS, stabilizing free radicals, and protecting cellular components from oxidative damage [37]. This activity contributes to the prevention of DNA mutations and the suppression of chronic inflammation, which are both implicated in carcinogenesis. However, in the tumor microenvironment or even in tumor cells, where redox balance is often perturbed, FA can paradoxically act as a pro-oxidant, further increasing intracellular ROS [32]. Indeed, tumors often produce a higher basal level of ROS, thereby displaying a lower threshold to oxidative stress condition [38]. This ROS overload, when further stimulated, can trigger mitochondrial dysfunction, caspase activation, and consequent apoptotic or autophagic cell death. For instance, FA treatment has been shown to induce a significant rise in ROS in MDA-MB-231cells, triggering mitochondria-mediated apoptosis through the activation of MAPK, STAT3, and NF-κB signaling pathways [39]. Thus, it can be generally stated that the redox-modulating activity of ferulic acid represents a central hub that integrates its chemo-preventive antimetastatic and therapeutic potential against cancer.

**Figure 2:**
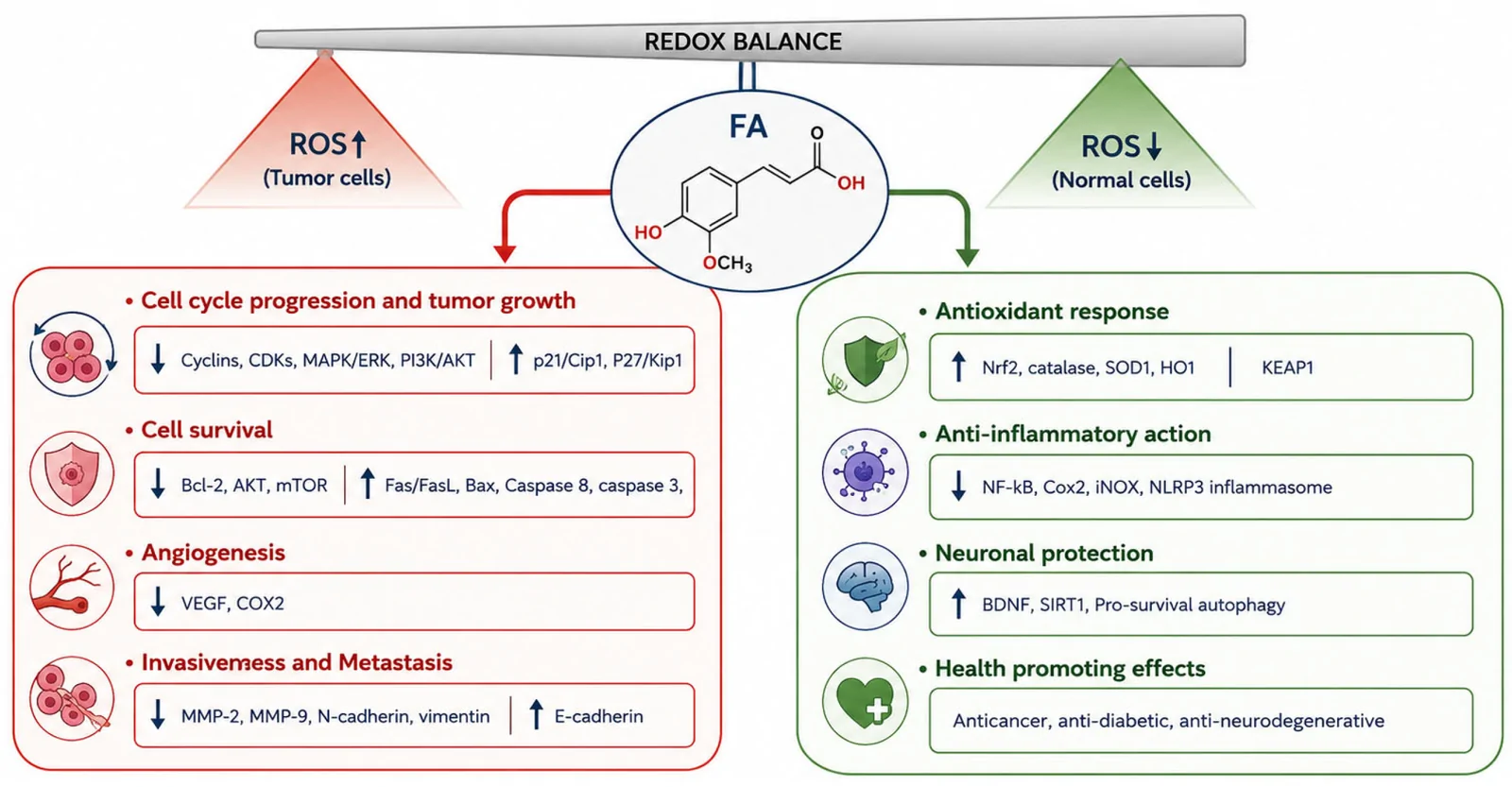
Anti-tumor properties of ferulic acid in relationship with its effects on cellular redox balance in normal and tumor cells: diversified effects and some factors involved. Illustration created by the Authors using AI assistance.

Anti-tumor properties of FA and its effects in non-tumor cells referred to the redox balance are summarized in Figure 2.

### 2.4. Combination of FA with Chemotherapeutics or Anti-Tumor Drugs

FA, as well as other derivatives of cinnamic acid, is also able to efficaciously act in combination with conventional chemotherapeutics such as cisplatin, paclitaxel, doxorubicin, and tamoxifen as described in a systematic review by Meirelles et al. [40]. An interesting approach combining FA with chemotherapeutics and using nanoparticles was recently provided by Helmy et al. who created doxorubicin/folate-targeted trans-ferulic acid-loaded poly lactic-co-glycolic acid (PLGA) nanoparticles combination for breast cancer treatment [41].

Apart from chemotherapeutics, other compounds have been used in combination with synergistic anti-tumor effects. For instance, evidence has been provided that a mixture of FA and p-coumaric acid suppresses colorectal cancer growth by metabolically interfering with aerobic glycolysis [42]. Resveratrol is another phenolic compound that displays a potent anti-tumor potential and that has been combined with FA [43]. Additionally, combination with poly-ADP ribose polymerase (PARP) inhibitors has been demonstrated to sensitize breast cancer cells to chemotherapy [11].

More recently, FA either alone or in combination with ascorbic acid attenuated cis-platin-induced ototoxicity [44,45].

Finally, in a very recent paper by Zhang et al., a cyclometalated iridium(III) complex incorporating FA was used as a promising metal-based programmed death-ligand 1 (PD-L1) inhibitor in lung carcinoma A549 cells [46]. Table 1 summarizes some anti-tumor compounds used in combination with ferulic acid.

**Table 1:** Some anti-tumor compounds used in combination with ferulic acid.

Chemotherapeutics				
	Treatment Conditions	Cell Line(s) or Animal Model(s)	Targeted Pathway	Refs
Cisplatin	In vivo	Male adult Wistar rats	ROS-modulated pathways	[45]
Paclitaxel	In vitro	Drug resistant human cervical adenocarcinoma cells	Proapoptotic factors activation	[47]
Doxorubicin	In vivo	Ehrlich carcinoma (EC)-bearing mice	Autophagic and apoptotic factors activation, oxidative stress reduction	[48]
5 Fluorouracil	In vivo	Colon tissues of male Wistar-albino rats	Apoptosis upregulation	[49]
**Other anti-tumor compounds**				
Veliparib (PARP inhibitor)	In vitro	Breast cancer cells	DNA repair reduction; DNA damage response increase	[11]
PD-L1 inhibitor	In vitro	Non-small cell lung cancer cells	Mitochondrial damage and autophagic cell death triggering; immune evasion block	[46]
Resveratrol	In vitro	Colon cancer cells	Loss of mitochondrial membrane potential and pro-apoptotic factors induction	[43]
p-coumaric acid	In vitro/in vivo	Human colorectal cancer cell lines/AOM/DSS mouse model	Growth suppression; aerobic glycolysis inhibition and reduced glucose consumption; lncRNA 495810 downregulation	[42]
Ascorbic acid	In vitro/in vivo	Mouse auditory hair cells and cochlear explants	Oxidative stress attenuation; cellular injury reduction; mitochondrial function preservation; MMP-9 and p38 MAPK-signaling suppression	[44]

## 3. Devices to Improve FA Bioavailability, Absorption and Anticancer Activity

Despite FA potential as anti-tumor agent, there are some limitations in the clinical applications. One of the major problems in FA-based anticancer therapy is its poor solubility in water and poor bioavailability, which is mainly due to its low absorption velocity in the gut, quite fast metabolism, and release from the body [50,51]. Pharmacokinetics studies of FA in humans have revealed that although it can be absorbed along the whole gastrointestinal tract, this occurs at a low rate because of its low ability to pass through biological membranes [2]. As concerns FA metabolism, the liver is the major organ involved, forming conjugated products with glucuronides and sulphate [52]. Subsequently, FA is excreted in urine as 3-hydroxyphenl and 3-methoxy-4-hydroxyphenyl derivatives of phenyl propionic acid and glycine conjugates [2].

To overcome these limitations, novel FA delivery strategies have been implemented, including the application of nano-formulations, polymer-based delivery systems, conjugation with specific chemical groups or biological compounds, leading to novel and promising FA derivatives. This paragraph describes the most significant tools to improve FA delivery and anti-tumor efficacy.

### 3.1. Conjugation of Ferulic Acid with Specific Carriers

#### 3.1.1. Nanoparticles

Over the last few years, several lines of evidence have indicated that encapsulating FA into nanoparticles results in efficacious drug delivery and improved anti-tumor effects. The types of nanoparticles used are quite heterogeneous and a plethora of combinations have been tested in tumor models with some satisfactory degree of activity. For instance, Rezaei et al. have shown that loading FA into cyclodextrin nanosponges improved the solubility up to fifteenfold compared with the pure compound and this exacerbated in vitro cytotoxicity in breast cancer cells [53]. Similarly, polymeric and lipidic nanocapsules of FA were prepared, characterized, and tested on colorectal cancer cell lines, where they displayed better anticancer activity than the compound alone [54]. In the context of lipid nanovectors, several formulations have been realized and tested in tumor models. Nanostructured lipid carriers were used to deliver FA in glioblastoma cells with promising results [55,56]. More recently, an interesting study by Miatmoko et al. has characterized the anticancer activity of PEGylated liposomes dually loaded with FA and doxorubicin (DOX). This study shows that co-loading FA into DOX liposomes enhanced cytotoxicity in cancer cells while displaying no effect on normal cells [57].

In addition, other types of nanovectors to deliver FA in tumor cells have been used. Alginate and chitosan-coated selenium nanoparticles were used to target breast cancer cells [58].

Polymeric mixed micelles of tocopheryl polyethylene glycol succinate loaded with FA displayed significant anticancer activity compared with FA alone in colon cancer cells via microRNA-221 mediated activation of tumor protein p53-inducible nuclear protein 1 (TP53INP1) [59].

The use of starch-based nanoparticles has been proposed to stabilize pickering emulsions to encapsulate FA and test them in cancer models with promising results [60].

Interestingly, Anbazhagan et al. have co-loaded FA and paclitaxel in co-loaded polyamidoamine dendrimers conjugated with arginyl-glycyl-aspartic acid to overcome P-glycoprotein-mediated multidrug resistance [47]. Moreover, in the context of targeted therapy, Zanin et al. have obtained efficient protein kinase CK2 inhibition by using a nanotechnological approach based on the use of surface-active maghemite nanoparticles. By coupling magnetism with photoluminescence, this approach provided direct visualization of the photoluminescent nanocarrier in tumor cells [61].

Nanotubes represent a promising vehicle for drug delivery. Therefore, this approach has also been considered to improve FA anti-tumor efficacy. An interesting study in this regard showed that combining FA with diosgenin loaded in multi-wall carbon nanotubes enhanced the anticancer effect of these drugs. These were evaluated in MCF-7 breast carcinoma cells, HepG2 hepatocellular carcinoma cells, and A549 non-small-cell lung cancer cells compared to normal human skin fibroblasts [62].

More recently, chitosan nanoparticles cross-linked by tripolyphosphate/Eudragit^®^ S100 were used as a colon drug delivery system to direct FA for the treatment of ulcerative colitis [63]. Although this study is not specifically focused on tumors, it must be considered that inflammation is a key hallmark of colon cancer, and it is worth mentioning this study since it provides valuable in vivo evaluation of FA delivery.

The above-mentioned approaches contribute to improving FA delivery and bioavailability. Overall, this leads to more efficacious anti-tumor action, which represents the best advantage of using nanodevices/nanovectors-based delivery systems. However, it must be considered that these strategies present some limitations that are specifically detailed in Table 2. One of the most frequent limits is the scarcity of translational/clinical applications exist in the literature, leaving the field wide open for further study. Nevertheless, some in vivo results are encouraging and sustain the translational potential of these delivery strategies. In this regard, it is worth mentioning the study by Riham I. El-Gogary et al. [54] who reported in vivo analysis in colon cancer revealing that FA lipid nanocapsules exhibited significant antioxidant and anti-inflammatory activities with specific apoptotic and anti-angiogenic actions, which were confirmed by histological examination. In addition, another representative in vivo study supported the efficacy of trans-ferulic acid (TFA) via loading into folate-receptor-targeted-poly lactic-co-glycolic acid nanoparticles (FA-PLGA-TFA NPs) and its association with doxorubicin for breast cancer treatment in rat models. Interestingly, this study also provided an in vivo safety profile of this combination through histopathological examination of the heart and bone, levels of hepatic enzymes in blood and leukocyte count [41].

**Figure 3:**
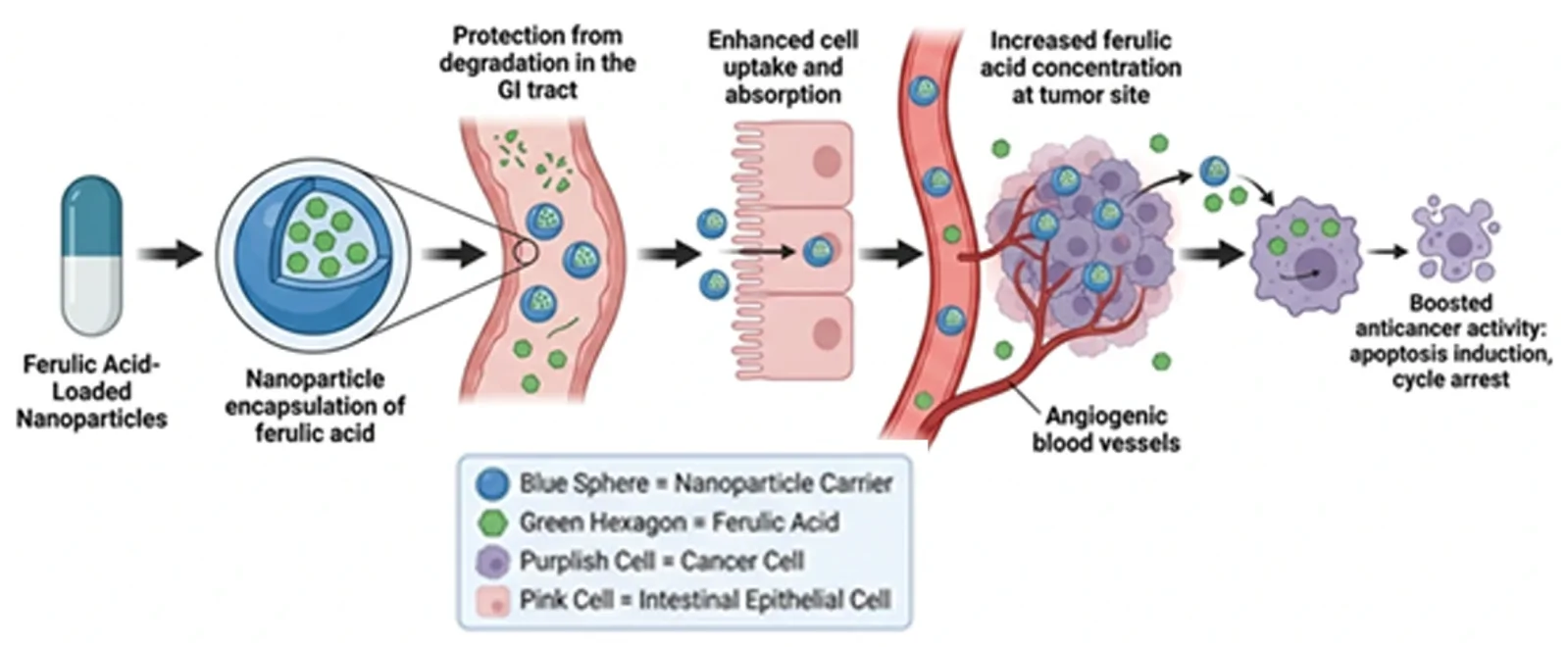
Schematic mechanism of nanoparticle-based delivery enhancing bioavailability and anticancer activity of ferulic acid. AI-generated illustration according to the indications of the Authors.

Overall, it is quite difficult to specify every single effect of the nanodevices used to properly deliver FA in the tumor site. However, in most cases, they improve intestinal absorption, targeted delivery and consequent apoptosis of cancer cells. The general mechanisms of increased bioavailability and anticancer activity of FA loaded into nanoparticles are described in Figure 3.

#### 3.1.2. Ferulic Acid Conjugation with Other Compounds

Another promising approach to improve FA anti-tumor effectiveness is to conjugate it with other compounds with known anticancer activity. Biotin has recently emerged as a good vehicle, in a conjugated form, to increase drug uptake, since its receptors are overexpressed in tumor cells [64]. Recently, evidence has been provided that conjugating FA with biotin significantly improved drug uptake by biotin receptor-positive tumor cells and efficaciously induced apoptosis [65].

Beta glucans are natural polysaccharides that display intriguing anticancer applications [66]. Li et al. have recently shown that feruloylated oat β-glucan exerts colon-targeted delivery, antioxidant and anticancer activity [67].

Metformin, a well-known drug for the therapy of type 2 diabetes, has been found to display significant effects in tumor growth inhibition and some metformin-based combinatorial therapies represent a promising tool [68]. Banerjee et al. have conjugated phenolic acids including FA, with metformin and studied their anti-tumor properties. These conjugates showed a good range of inhibitory effects in tumor cells [69].

Another interesting conjugation approach was used for breast cancer targeted therapy. In this case, FA was conjugated with geldanamycin, a well-studied heat shock protein 90 (HSP90) inhibitor. Considering the pro-survival and pro-tumoral role of HSP90, it was relevant that the conjugated compound was more efficacious than 17-AAG (a geldanamycin derivative) alone in vivo, exhibiting a lower profile of hepatic toxicity [70].

In the context of FA derivatives, novel and recent insights were provided by Mohie et al. who designed and synthesized seven novel FA derivatives with chemically masked hydroxyl (-OH) groups [71]. Notably, these compounds were tested in hepatocellular carcinoma cells, where they displayed potent anti-tumor activity while showing a safety profile in normal fibroblasts. Specifically, one of these derivatives, named compound 4e, revealed potent apoptotic and anti-angiogenic effects targeting vascular endothelial growth factor receptor (VEGFR-2).

Evidence has also been provided that FA can be conjugated with triphenylphosphonium salts (TPP) with both amphiphilicity and tumor mitochondrial targeting activity. Specifically, the manuscript by Wu et al. shows a particular anticancer efficacy of TPP-conjugated FA derivatives both in vitro and in vivo [72].

Finally, a simpler modification of FA structure was introduced by hydroxylation producing 5-hydroxyferulic acid (5-OHFA), which was characterized in vitro and in silico for biological properties and cytotoxic potential [73].

### 3.2. Chemical Features of Ferulic Acid Derivatives and Their Anti-Tumor Properties

To further describe FA derivatives, it is important to consider the chemical features of the compound and potential interactions with ligands that can potentiate its anti-tumor efficacy. Therefore, the following paragraphs go into more detail about the chemical properties of the molecule and interactive networks.

#### 3.2.1. Metal Complexes of Ferulic Acid

Structurally, FA belongs to the cinnamic acid derivatives. The precursor cinnamic acid has significant biological activity and distinctive structure that makes it a widely studied compound. The molecule contains a double bond between the aromatic ring and the carboxylic group, causing conjugations between the carbon-carbon double bond and the pi-electron system. Substituting metal ions within the carboxylic acid group of cinnamic acid and its related compounds leads to alterations in the distribution of electrons throughout the entire molecule. The specific way the metal ion binds to the carboxylate group causes these changes in its electronic structure. Metal ions primarily influence the electron distribution around the double bond atoms of cinnamate and its derivatives, with a less significant impact on the aromatic ring’s electronic structure. The size of the metal ion, specifically its ionic radius, is the main factor determining the interaction between the metal ion and the cinnamic acid molecule [74]. Studies indicate that the hydroxyl groups on their aromatic structure are crucial for their antioxidant activity. These phenolic compounds typically act as antioxidants by scavenging radicals through hydrogen or electron donation, leading to stable phenoxyl radicals. They may also counteract oxidative stress by chelating transition metals such as copper and iron [75,76].

The impact of zinc, cadmium(II), and mercury(I) ions on the electronic structure of cinnamic acid (phenylacrylic acid) was explored by Kalinowska et al. using a combination of complementary analytical methods: Fourier transform infrared (FT-IR), FT-Raman, nu-clear magnetic resonance (1H and 13C NMR) spectroscopies, and quantum mechanical calculations [74].

Hydroxycinnamic acids have been extensively studied for their antioxidant and anti-cancer potential [77,78]. For instance, five new [Ru(L)(dppb)(bipy)]PF6 complexes, where L = cinnamic acids or derivatives synthesized, were characterized and their cytotoxicity activities were evaluated in MDA-MB-231 and MCF-7 breast cancer cells and in the non-tumor MCF-10A and L929 cells. Complexes [Ru(L4)(dppb)(bipy)]PF6 (L4 = 4-hydroxycinnamic acid) and [Ru(L5)(dppb)(bipy)]PF6 (L5 = 3,4-dihydroxycinnamic acid) were the most selective in tumor cells, presenting high selectivity indices since they inhibited some processes related to tumor progression, such as invasion, migration, and adhesion of triple negative MDA-MB-231 cells [79].

FA possesses functional groups like carboxylic acid and phenolic hydroxyl groups, which can act as ligands to coordinate with metal ions. The planar structure of FA stabilized by pi-electron delocalization, positively impacts its free radical scavenging capacity. Four antioxidant mechanisms important in free radicals scavenging have been studied by Urbaniak et al.: Hydrogen atom transfer (HAT), sequential proton loss electron transfer (SPLET), single electron transfer–proton transfer (SET-PT) and transition metal chelation (TMC) [75].

In physiological conditions (pH = 7.4), FA is present mostly in the form of mono-anion (HA−) along with its neutral form (H2A) [80].

FA can bind to various metal ions, including transition metals like zinc Zn(II), cadmium Cd(II), mercury Hg(I), copper Cu(II), iron Fe(III), nickel Ni(II), cobalt Co(II), aluminum Al(III), and alkali metals like sodium Na(I) and magnesium Mg(II). The resulting complexes can have different stoichiometries (metal-to-ligand ratios) and structures, including mononuclear and polynuclear species. The formation of metal complexes can lead to shifts in the IR, Raman, and nuclear magnetic resonance (NMR) spectra of ferulic acid, providing information about the coordination environment and the electronic structure of the complexes. The stability of these complexes varies depending on the metal ion and the nature of the bonding. Stability constants can be determined using techniques like potentiometric titration [81].

Complexation with certain metal ions can modulate the antioxidant activity of ferulic acid. In some cases, the metal complex might exhibit enhanced or altered radical scavenging properties compared to the free ligand. Metal complexation might also influence other biological activities of FA, such as its enzyme inhibitory capacity or anticancer activity. For instance, evidence has been provided that complexing FA to metals strongly increases the toxicity of the obtained compound in immortalized human keratinocytes [82].

To the best of our knowledge, few studies in the literature are dedicated to the anti-tumor action of FA-metal complexes. However, the chelating biological activity of FA has been widely associated with its anti-tumor properties. Indeed, it has been shown that radical scavenging and ferrous ion chelating activities of FA account for anti-cancer and pro-apoptotic actions [83]. Specifically, the antioxidant activity of FA associated with chelation with ferrous ions is known to prevent reactive hydroxyl radical formation via Fenton or Fenton-like reactions. All the complexation processes were computed in the implicit aqueous solution to mimic influence of physiological environment. It is noted that the deprotonation site for the mono-anionic form of FA (HA−) is determined by the carboxylic group (COOH). In addition, both the neutral and mono-anionic forms of FA can chelate Fe(II) ion via monodentate or bidentate coordination. Computational analyses were performed to understand the reduction of iron(III) complexes to iron(II) using superoxide (O2−) and ascorbate (Asc−) anions. These calculations showed HA− is the preferred species for chelating both iron ions, compared to its neutral form (H2A). Moreover, the binding reactions with iron(III) were calculated to be more energetically favorable than those with iron(II). The high pro-oxidant risk of FA resulted from the formation of Fe(II) ions which induce the production of hydroxyl radicals by Fenton or Fenton-like reactions. The antioxidant activities of FA are generally more preponderant than the pro-oxidant risk, although the latter becomes considerable in some specific conditions, like high concentration of FA.

Nevertheless, the pro-oxidant activity of FA can also be exploited to target tumor cells. Specifically, concerning cellular iron content and metabolism, some controversial data are present in the literature. For instance, Cao et al. have recently shown that FA exposure in esophageal squamous cell carcinoma cells led to ROS production and iron load with consequent ferroptosis [32].

FA is best known as an antioxidant at low concentrations, whereas at higher concentrations or in the presence of metal ions, it may act as a pro-oxidant. Another evidence of pro-oxidant and anti-tumor activity of FA was previously provided by Sarwar et al. who demonstrated that copper enhanced the ability of FA to induce ROS generation and consequent cell death in tumor cells [84].

Overall, metal complexes of ferulic acid could potentially be used to enhance the bioavailability or stability of both the metal ion and the phenolic acid. However, despite several chemical approaches to coordinate FA with metal ions, including magnesium, zinc and copper, and explore their biological activities, to date no data are present in the literature about their anti-tumor properties.

#### 3.2.2. Organotin(IV) Derivatives of Ferulic Acid

Organotin(IV) compounds represent a class of non-platinum metal-pharmaceuticals exhibiting a promising anti-tumor activity [85]. We have recently described the synthesis and chemical characterization of new organotin(IV) complexes with epigenetic modulator ligands and their impact in cancer therapy [86].

Historically, since 1997 Willem proposed organotin(IV) derivatives of cinnamic acid, the ferulic acid precursor, for anti-tumoral properties. He synthesized triphenyltin and tri-n-butyltin cinnamates in order to compare their chemical properties and antitumour activities with those of the corresponding pentafluorophenyl analogs. The study assessed the influence of substituting a phenyl group for a pentafluorophenyl one in a series of triorganotin carboxylates. The results of the in vitro anti-tumor screening of the triorganotin carboxylates against a selected panel of six human tumor cell lines, (MCF-7 and EVSA-T, two breast tumors, WiDr, a colon carcinoma, IGROV, an ovarian cancer, MI9 MEL, a melanoma, and A498, a renal cancer) showed that tri-n-butyltin compounds are significantly more active or at least of the same order of activity against all cell lines when compared with carboplatin, cis-platin or 5-fluorouracil. In particular, the tri-n-butyltin and triphenyltin cinnamates were especially active against EVSA-T [87].

More recently, some new organotin(IV) hydroxycinnamates, including an organotin(IV) complex of ferulate, were designed, synthesized and characterized for their in vitro cytostatic activity against HeLa cell lines and HepG2 cell lines [88].

**Figure 4:**
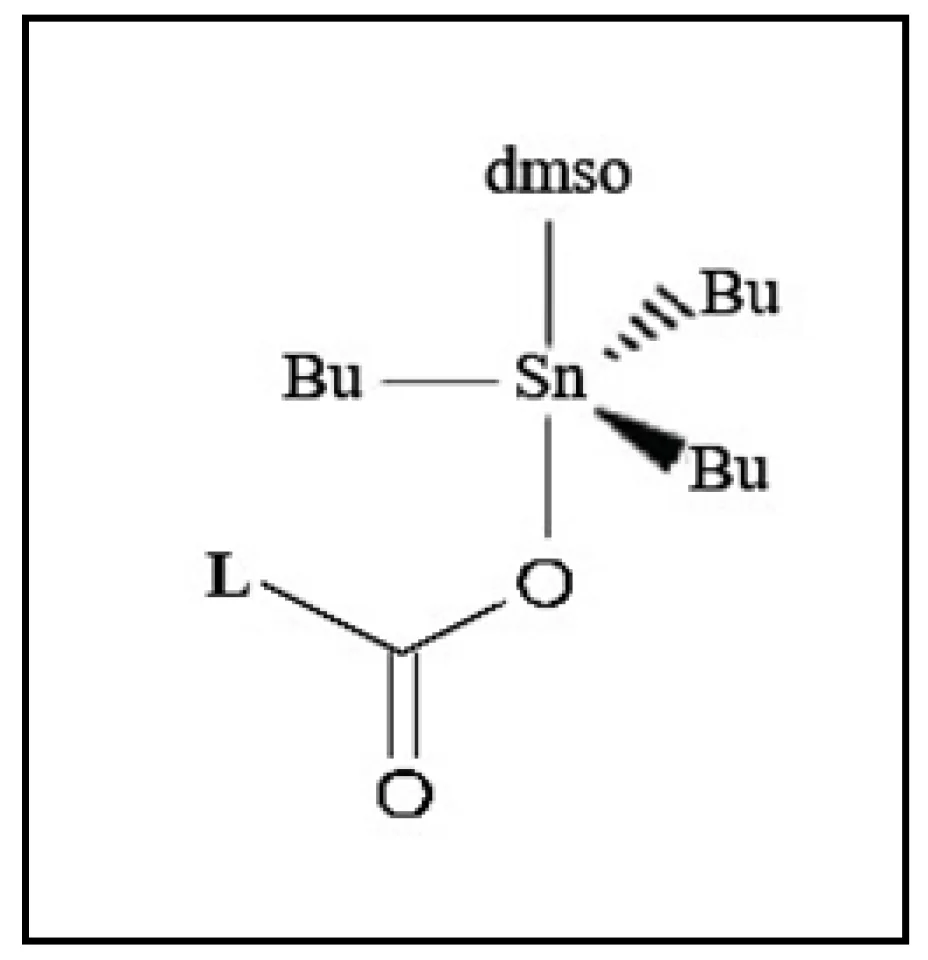
Tributyltin(IV) ferulate structures in coordinating DMSO-d6 solvent.

As specifically concerns FA, tributyltin(IV) ferulate (TBT-F) is an organotin derivative with potent anti-tumor activity. This compound was specifically synthesized with the aim of enhancing the anticancer potential of FA [89]. In terms of coordination chemistry, FA is a potential bidentate monoanionic ligand exerting its coordinating power toward the Lewis acids, indeed the carboxylate oxygen atoms (COOH) and the hydroxyl group of the phenolic ring (OH) or may act as donor atoms. Therefore, by infrared spectroscopy, evidence has been provided that the tin atom in this derivative is at least five-coordinate, with the ligand acting as a monoanionic bidentate ligand through a bridging carboxylate anion [89,90,91]. The pentacoordinated structure of TBT-F in dimethyl sulfoxide (DMSO) solvent is illustrated in Figure 4.

The anti-tumor effects of TBT-F have been evaluated in colon cancer cells [89]. Interestingly, this paper showed that TBT-F significantly reduced the viability of three colon cancer cell lines (HCT116, Caco-2 and HT-29) at nanomolar concentration range. In contrast, the parental compound tributyltin(IV) chloride (TBT) exhibited only limited cytotoxicity whereas FA was completely inefficacious at the same treatment conditions.

The biochemical characterization revealed that TBT-F did not induce apoptotic or necrotic cell death but rather autophagy. Subsequently, the precise mechanism was described in HCT116 colon cancer cells, where the compound induced ROS production, endoplasmic reticulum (ER) stress and autophagic cell death [92]. Therefore, the organotin moiety confers pro-oxidant properties to FA even at low concentrations. Moreover, the reduced toxicity of TBT-F compared to the parental TBT, renders the molecule a good candidate for colon cancer treatment.

Notably, halloysite nanotubes (HNT) found applications in microbiology as an antimicrobial agent [93,94]. Combining TBT-F with nanocarriers can represent a promising strategy to enhance its biological activity, further reducing toxicity. With this purpose, a functional biohybrid material was developed by loading TBT-F into HNT and is currently under evaluation in tumor models [95,96].

In the context of drug delivery in colon cancer, an interesting employment of FA was provided by Zheng et al. who created polycondensed-ferulic acid (PFA) to serve as an efficacious drug carrier with anticancer potential [97].

Overall, considering the low water solubility and instability of FA in the colon, the ferulic organotin derivative TBT-F, with its stability and ability as a pro-oxidant, represents a promising drug for colon cancer treatment.

## 4. Conclusions

The new findings about how FA can be chemically conjugated with other compounds or incorporated into nano-carriers open new perspectives on its therapeutic use against cancer. Different approaches to improve FA bioavailability and water solubility have been presented in this review providing several examples of increased anti-tumor efficacy of FA derivatives, chemical conjugates or specific delivery nanodevices. However, it must be emphasized that all these strategies present advantages and limitations that would be difficult to discuss case by case. Therefore, the summary in Table 2 provides detailed information on each category. Since the most frequent limitation of the numerous approaches reported in this review is represented by a lack of translational/clinical data in the literature and only a few in vivo data are present in the literature, the current challenges and future prospects include: (1) Strengthening the in vitro results with more in vivo evaluations; (2) Performing safety profile analysis of the different approaches for clinical application.

**Table 2:** Strategies to improve the anti-tumor efficacy of ferulic acid: main objectives, advantages, and limitations.

Approach	Main Objective	Advantages	Limitations/Challenges
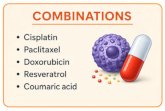	**Enhance therapeutic efficacy and overcome resistance**	• Synergistic or additive anticancer effects. • Potential reduction in the dose of conventional chemotherapeutics. • Simultaneous modulation of multiple molecular pathways. • Potential to overcome or delay drug resistance.	• Synergy depends strongly on drug ratio, dose, and treatment schedule. • Possible increased toxicity due to drug–drug interactions. • Pharmacokinetic and pharmacodynamic interactions may complicate treatment optimization. • Molecular mechanisms underlying synergy are often incompletely characterized.
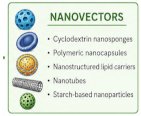	**Improve** **bioavailability, stability, tumor targeting, and controlled delivery**	• Improve FA solubility, stability, and bioavailability. • Protect FA from premature degradation and metabolism. • Facilitate preferential accumulation in tumor tissue. • Enable controlled or sustained release. • Allow co-delivery of FA with other anticancer agents.	• Potential toxicity and immunogenicity of nanomaterials. • Complex formulation and scale-up procedures. • Variable drug loading and release profiles. • Limited knowledge of long-term biodistribution and clearance. • Regulatory and manufacturing challenges.
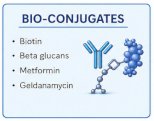	**Increase tumor selectivity and cellular uptake**	• Covalent conjugation to peptides, proteins, polymers, or other biomolecules can improve pharmacokinetic properties. • May enhance cellular uptake and tumor selectivity. • Can exploit receptor-mediated targeting mechanisms. • May improve FA stability and prolong its biological activity.	• Conjugation may reduce the availability of the active FA moiety. • Requires controlled and reproducible synthesis. • Potential immunogenicity or altered biological activity. • Limited in vivo validation for many FA-based conjugates.
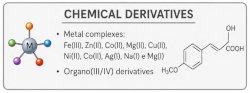	**Increase intrinsic potency and optimize physicochemical properties**	• Can improve lipophilicity, solubility, stability, and cellular permeability. • May enhance intrinsic anticancer potency. • Enables structure–activity relationship (SAR) optimization. • Can generate derivatives with improved tumor-cell selectivity. • May overcome the relatively low potency of native FA.	• Structural modifications may alter FA’s antioxidant and biological properties. • Increased potency may be associated with increased toxicity. • Synthesis may require multiple chemical steps and extensive purification. • Metabolic stability and pharmacokinetic behavior may differ from native FA. • Extensive preclinical and toxicological validation is required for clinical translation.

## Data Availability

No new data were created or analyzed in this study. Data sharing is not applicable to this article.
